# The incidence of lung cancer amongst primary care chest radiograph referrals—an evaluation of national and local datasets within the United Kingdom

**DOI:** 10.1093/bjr/tqae142

**Published:** 2024-08-20

**Authors:** Bobby S K Bhartia, David Baldwin, Stephen H Bradley, Matthew E J Callister, Indrajeet Das, Matthew Evison, Seamus Grundy, Jaspreet Kaur, Martyn Kennedy, Emma L O’Dowd

**Affiliations:** Department of Clinical Radiology, Leeds Teaching Hospitals NHS Trust, Leeds, LS9 7TF, United Kingdom; Respiratory Medicine, Nottingham University Hospitals NHS Trust, University of Nottingham, NG5 1PB, United Kingdom; School of Medicine, University of Leeds, Leeds LS2 9JT, United Kingdom; Department of Respiratory Medicine, Leeds Teaching Hospitals NHS Trust, Leeds, LS9 7TF, United Kingdom; Department of Clinical Radiology, University Hospitals of Leicester NHS trust, Leicester, LE1 5WW, United Kingdom; Wythenshawe Hospital, Manchester University NHS Foundation Trust, Manchester, M23 9LT, United Kingdom; Department of Respiratory Medicine, Salford Royal Hospital, Northern Care Alliance NHS Foundation Trust, Salford, M6 8HD, United Kingdom; University of Nottingham, Nottingham, Nottingham, NG7 2RD, United Kingdom; Department of Respiratory Medicine, Leeds Teaching Hospitals NHS Trust, Leeds, LS9 7TF, United Kingdom; Respiratory Medicine, Nottingham University Hospitals NHS Trust, University of Nottingham, NG5 1PB, United Kingdom

**Keywords:** lung cancer, incidence, primary care, chest X-ray, diagnostic accuracy

## Abstract

**Objectives:**

To determine the incidence of lung cancer amongst primary care referrals for investigation with a chest radiograph (CXR).

**Methods:**

Retrospective evaluation of datasets from the national Clinical Practice Research Datalink (CPRD) and from a single large regional centre. Data were extracted for cohorts of consecutive adults aged over 40 years for whom a CXR had been performed between 2016 and 2018. Using cancer registry data, the incidence of lung cancer within a 2 years of the CXR referral and the variations with age, gender, and smoking status were evaluated.

**Results:**

A total of 291 294 CXR events were evaluated from the combined datasets. The incidence of lung cancer amongst primary care CXR referrals was 1.4% in CPRD with a consistent correlation with increasing age and smoking status. The incidence of lung cancer within two-years of the CXR varied between 0.03% (95%CI, 0.0-0.1) amongst never smokers aged 40-45 years to 4.8% (95%CI, 4.2-5.5) amongst current-smokers aged 70-75 years. The findings were similar for the single large centre data, although cancer incidence was higher.

**Conclusions:**

A simple estimation and stratification of the risk of lung cancer amongst primary care referrals for investigation with a CXR is possible using age and smoking status.

**Advances in knowledge:**

This is the first estimate of the incidence of lung cancer amongst primary care CXR referrals and a demonstration of how the demographic information contained within a request could be used to optimize investigations and interpret test results.

## Introduction

The chest radiograph (CXR) is the initial radiological investigation recommended in the majority of individuals with symptoms of potential lung cancer.^1^^,^^2^ Further investigation for lung cancer, typically with CT is advised following an abnormal CXR result. Although the CXR is a safe and widely available test, the test has a limited sensitivity of between 77% and 81% for the detection of lung cancer.^3^

The majority of patients with lung cancer initially present via their general practitioner.^4^ The clinical diagnosis is difficult due to the low prevalence of disease and symptoms which are often non-specific or possibly co-incidental.^5^ Amongst the primary care population less than 1 in 200 patients with a persistent cough which is the most frequent symptom in lung cancer will be eventually diagnosed with lung cancer. It is estimated that in 17%-34% of cases of diagnosed lung cancer, the symptoms were unrelated to the diagnosis.^6^ Clinicians are encouraged to have a low threshold for investigation.

The demonstration that screening asymptomatic individuals with the more sensitive low-dose CT (LDCT) can reduce both lung cancer and all-cause mortality has generated interest in whether LDCT might have a role as the initial investigation in those presenting with symptoms.^7^^,^^8^ In the United Kingdom, the Health Technology Assessment programme is currently evaluating the feasibility of replacing the CXR with LDCT as a first line investigation for lung cancer.^9^ An understanding of the incidence of lung cancer amongst those currently referred for investigation, and how this might vary with characteristics such as age, gender, and smoking status, is a pre-requisite for considering changes to the imaging pathway. We anticipate that the incidence of lung cancer amongst those referred for investigation would differ and likely be higher than that found in the general or primary care population due to the presence of symptoms and the selection of patients for referral by the general practitioner.

We evaluated referrals from primary care identified within the national Clinical Practice Research Datalink (CPRD) and referrals to single large secondary care service in the north of England. Due to the non-specific nature of the symptoms of lung cancer, the study determined the incidence of lung cancer amongst referrals for investigation with a CXR for all indications. The incidence was determined using cancer registry information to identify those diagnosed with cancer within the 2-year period of the referral for investigation. This study did not examine the outcomes or influence of the CXR examination.

## Methods

We performed 2 separate retrospective evaluations of the electronic records of a national dataset (CPRD) and of the Radiology Information System (RIS) of Leeds Teaching Hospitals NHS Trust (LTHT). CPRD is a computerized primary care database linked to a range of other health-related data to provide a representative UK population health data set. The longitudinal data encompass 50 million patients, including 14 million who are currently registered.^10^ LTHT is a large secondary and tertiary care centre in the north of England and serves an urban region with over 93 primary care practices and 866 000 registered patients and a high incidence of lung cancer. The 2 evaluations allowed us to determine if the results obtained from a large dataset representative of national data would be comparable to local data extracted from a different source. The local data had the advantage of a contemporary smoking status being recorded at the time of the request for a CXR.

### Data extraction

Both CPRD and the LTHT RIS were searched for all CXRs referred from primary care and performed in adults aged 40 years or older between April 1, 2016 and March 31, 2018. The dates were selected to allow for 2-years of follow-up before the onset of the COVID-19 pandemic in March 2020 disrupted diagnostic services within the United Kingdom. Referrals for all indications were included because of the weak association between symptoms and the diagnosis of lung cancer. The information retrieved included the age, gender, and smoking status. Smoking data in CPRD are recorded whenever the person visits the GP. The GP records the details using medical and READ codes as “current-,” “ex-,” or “never-.” In LTHT RIS, there is a prompt to categorize smoking status upon requesting CXR as “current,” “ex-smoker,” “never smoker,” and “unknown.”

Patients who were diagnosed with lung cancer (International Classification of Diseases ICD-10 C34) were identified from CPRD using medical, SNOMED CT and READ codes. The date of the decision of the multidisciplinary team (MDT) was used as the diagnosis date and the difference between the date of the CXR examination and MDT decision calculated for each examination. Diagnoses within 730 days were considered evidence of the presence of cancer.

To ensure that cases were incident rather than prevalent cases within the CPRD data, we excluded patients who registered <12 months prior to their diagnosis date. This method prevents patients with established diagnoses who move location being mistaken for a new diagnosis when their clinical details are coded at a new practice and has been previously utilized in epidemiological studies using the CPRD.^11^^,^^12^ In addition, if the diagnosis date predated the CXR examination date the study was excluded from the analysis. The cases retrieved from LTHT RIS were cross referenced with the local cancer registry using the same ICD-10 and date analysis.

### Analysis

The incidence of lung cancer within 2 years of the CXR referral was calculated for 5 years age bands between 40 and 80 years with 95% CI according to gender and smoking status.

The incidence was estimated as the incidence per CXR examination performed and per individual. The incidence per examination is useful when interpreting the results of a single CXR event but may overstate the incidence within the population if the presence of cancer were associated with undergoing multiple examinations. For the incidence per individual, the first CXR performed was used as the reference test when more than one examination was performed within the 2-year cohort sampling period. The first episode was selected as the results would reasonably reflect the impact of referral for alternative testing with CT at the first presentation.

As an illustration of the potential value of the data, an estimate of the proportion of the examinations with an incidence of lung cancer greater than 3% was made to determine the potential impact of applying this threshold in the investigation of suspected lung cancer. The threshold is referenced in National Institute for Health and Care Excellence (NICE) guidelines on investigations for cancer above which patients should be referred directly for more extensive evaluation and represents a potential threshold for directing patients to evaluation with CT.

Chi-squared testing was used to evaluate the differences in proportions between categorical variables.

## Results

### Patient imaging characteristics

About 228 855 and 62 439 CXRs were identified from CPRD and LTHT-RIS, respectively. The demographic background associated with the investigations is outlined in Table 1.

**Table 1.** Characteristics of the population on a per examination basis.

	CPRD				
	Total	Current-smoker	Ex-smoker	Never-smoker	Unknown
Examinations (% of examinations)	228 855 (100)	52 051 (22.7)	101 460 (44.3)	67 722 (29.6)	7622 (3.3)
Lung cancers identified within 2-yrs	3205	1273	1697	179	56
Incidence % (95% CI)	1.40 (1.35-1.45)	2.45 (2.31-2.58)	1.67 (1.59-1.75)	0.26 (0.23-0.31)	0.73 (0.56-0.95)
Female (%) Median age in years (interquartile range)	52.3 67 (56-76)	52.7 61 (52-70)	47.9 70 (60-78)	60.8 65 (55-76)	52.8 66 (54-78)
	LTHT-RIS				
Examinations (%)	62 439 (100)	11 798 (18.9)	21 804 (33.8)	24 410 (39.8)	4430 (7.5)
Lung cancers identified within 2 years	968	340	456	99	73
Incidence % (95% CI)	1.55 (1.45-1.65)	2.88 (2.58-3.21)	2.09 (1.90-2.29)	0.41 (0.33-0.49)	1.65 (1.29-2.07)
Female (%) median age in years (interquartile range)	54 64 (54-74)	54.7 57 (50-67)	46.5 69 (58-77)	60.6 64 (53-74)	52.1 65 (56-73)

**Figures 1.**
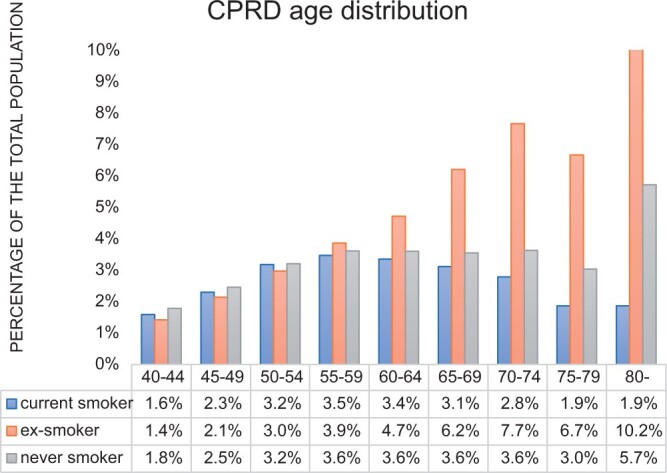
The age distribution amongst Clinical Practice Research Datalink (CPRD) examinations of different smoking status.

**Figures 2.**
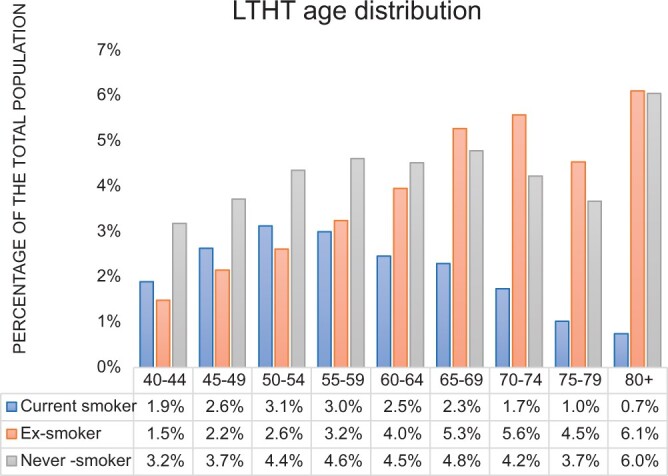
The age distribution amongst LTHT-RIS examinations of different smoking status.

The proportion of each smoking status differed between the CPRD and LTHT-RIS datasets with a higher proportion of never-smokers amongst the LTHT-RIS cases (*P* < .001) (Table 1). For each smoking category, the distribution of age and gender were similar between the 2 datasets (Figures 1 and 2). In both groups, the most frequent age for imaging current-smokers was 50-59 whilst for ex-smokers imaging tended to occur at older ages, with the most frequent ages being 70-74 years and ≥80 years.

### The incidence of lung cancer

The overall incidence of lung cancer within 2 years of the CXR examination was 1.40% in the CPRD and 1.55% in the LTHT-RIS data (*P* = .0052). A table of the full results is provided in Supplementary Appendix S1 and S2.

**Figure 3.**
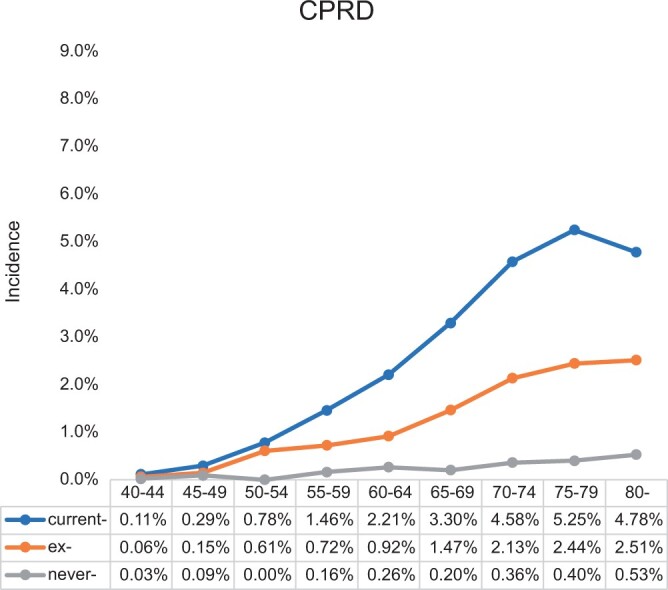
Incidence of lung cancer within 2 years of a chest radiograph (CXR) examination by age and smoking status from national Clinical Practice Research Datalink (CPRD).

**Figure 4.**
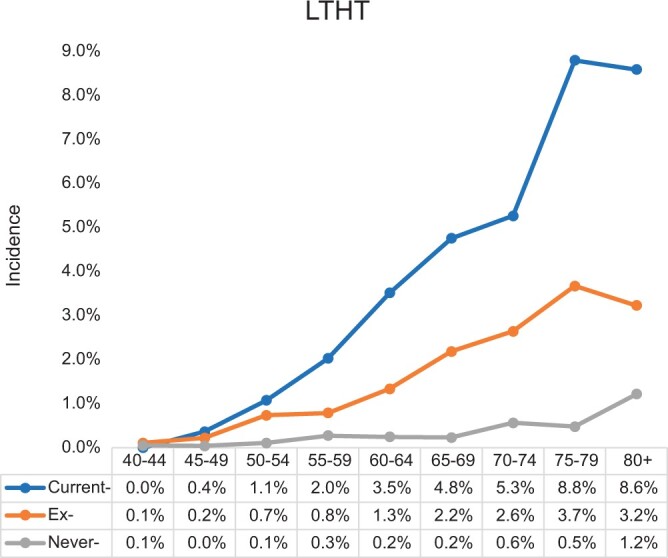
Incidence of lung cancer within 2 years of a chest radiograph (CXR) examination by age and smoking status from LTHT.

Within both national and local datasets, the peak incidence occurred in current-smokers aged 75-79 years (Figures 3 and 4). Amongst ex- and never-smokers there was a progressive increase in incidence with increasing age.

Compared to the CPRD, the LTHT-RIS data demonstrated a higher incidence especially within current- and ex-smokers (*P* = .006 for current smokers and *P* < .001 for ex-smokers).

The incidence per individual was also estimated (Supplementary Appendix S2). The results demonstrate a similar progressive increase in lung cancer incidence with increasing age and increased smoking related risk with the peak incidence amongst current-smokers aged 75-79 years.

There was a statistically significantly higher incidence of per examination compared to per patient within the CPRD dataset (1.4% vs 1.3%, *P* = .0035) which suggests that the diagnosis of lung cancer is associated with undergoing an increased number of CXR examinations. A statistical significance was not demonstrated within the smaller LTHT-RIS dataset.

Patient sex did not influence the incidence of lung cancer per CXR examination when corrected for differences in smoking prevalence (Current-smokers *P*-value .47, Ex-smokers *P*-value .06, Never-smokers *P*-value .63) (Supplementary Appendix S3).

**Figure 5.**
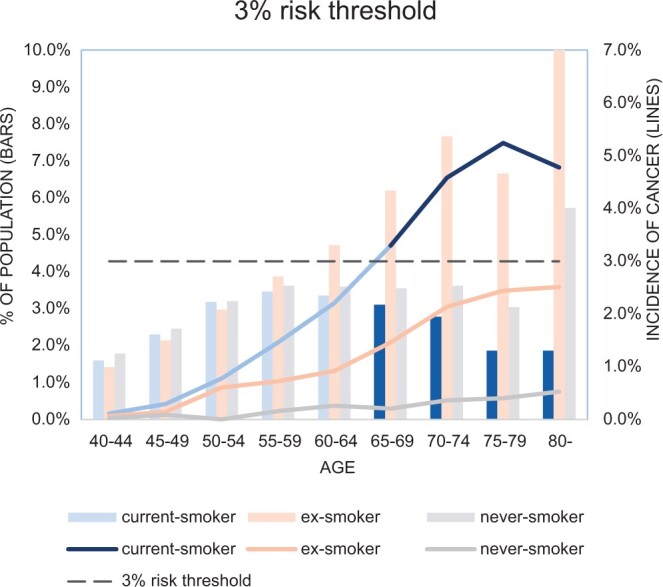
Line graph of the incidence of lung cancer with the proportion of the population for differing age and smoking categories. The dashed line indicates a 3% risk threshold for lung cancer. Current-smokers aged over 65-69 have an incidence above 3% (dark columns). Applying this threshold to alter the initial investigation (to CT) would apply to 9.7% of all current chest radiograph (CXR) referrals.

The proportion of the examinations within the CPRD population with an incidence of lung cancer >3% was determined by combining the incidence of cancer and the histogram of the distribution of the population and estimated to be 9.7% of CXRs (Figure 5).

## Discussion

This study is the first evaluation of the risk of lung cancer amongst those referred for investigation with a CXR from primary care in the United Kingdom. The larger of the 2 datasets demonstrates that the overall incidence of lung cancer amongst CXR referrals is approximately 1.4% and the risk of underlying cancer can be stratified effectively using patient age and smoking status. The study also demonstrates that the stratification of the risk of cancer can be reproduced at a local level in at least one centre, albeit with a higher overall incidence.

Due to the concerns around the limitations of the CXR direct access to CT from primary care is proposed and available in some areas, yet it is not clear how or when to select patients for CT in the presence of a normal CXR. It is unlikely that there will be sufficient radiology capacity to investigate all symptomatic patients with CT due to the low predictive value of symptoms. Recognizing those at the greatest risk is clinically challenging. Extensive prior research into the clinical presentation of lung cancer indicates that the clinical history may be unhelpful as the presenting symptoms and signs of lung cancer are frequently non-specific.^2^ The most common symptoms reported amongst those with lung cancer are unexplained cough and dyspnoea. These occur frequently within the population resulting in a low positive predictive value (PPV) of 0.4% (95% CIs 0.3-0.6) and 0.66% (95% CIs 0.5-0.8), respectively.

This evaluation shows that simple stratification based on age and smoking status could provide a rationale for who to prioritize for direct access to CT. As an illustration, the NICE advise a 3% PPV of signs and symptoms as a threshold for urgent referral for further evaluation of suspected cancer. They recommend further evaluation in the presence of an abnormal CXR or unexplained haemoptysis. Unexplained haemoptysis as a single symptom exceeds the 3% threshold, and the level of risk is great enough to warrant automatic urgent referral for further investigation rather than a CXR, but the symptom is encountered in <5% of presentations.

The study demonstrates that referral for a CXR amongst certain demographic groups similarly reflects a 3% risk of underlying cancer. The findings indicate that nationally referrals amongst current-smokers over the age of 65-years exceed a 3% threshold and this could be used as the rationale for routine further evaluation beyond the CXR. These would account for approximately 9.7% of CXR referrals from primary care (Figure 5). The results from the single centre broadly corroborate the national data but also indicate that the age and smoking thresholds may prove to be wider in populations with a higher underlying risk of cancer.

The limited sensitivity of the CXR is well recognized.^3^^,^^13^ An understanding of the pre-test probability is an essential component of interpreting the significance of a CXR test result. The post-test probability can be determined by applying the likelihood ratio of a negative test to the incidence of cancer. Previous studies of diagnostic accuracy have determined the LR of a negative CXR to be 0.4 for the diagnosis of lung cancer within 2-years of a CXR.^14^ When applied to a current-smokers aged 75-79 years with a pre-test probability of 5.25%, the post-test probability of developing lung cancer within 2 years of following a negative CXR would be remain ∼2.2%. Conversely, the highest pre-test-probability amongst never-smokers is 0.53% indicating that probability of lung cancer within 2-years of a negative CXR is 0.2% and is unlikely to routinely warrant further investigation.

A detailed understanding of pre-test probability may also prove to be of use in developing new tools for the evaluation of the CXR within the rapidly developing field of artificial intelligence. These algorithms determine the probability of the presence of an abnormality following analysis of the CXR image and apply varying thresholds to classify a CXR as normal or abnormal. The reliability of this automated decision making may be greatly improved if the prior probability of cancer can be incorporated and the threshold for a positive test altered using simple metrics such as the age and smoking-status.

Although the CXR remains the recommended first line investigation for the majority of people with symptoms of lung cancer the National Institute for Health and Care Research is currently commissioning research to determine the feasibility of using low dose CT as an initial investigation (NIHR 22/98). This work also provides an indication of the likely diagnostic yield of applying such a policy and a potential means to optimize the cost effectiveness of such a strategy by applying simple risk thresholds based on age and smoking status to triage patients to low-dose CT (Figure 5).

## Study strengths and limitations

The study evaluates two large independent sets of data and demonstrates how the underlying risk of lung cancer can be stratified using the patients age and smoking status. Other factors which influence the risk of underlying cancer include the prevalence of disease, patient health seeking behaviour and the willingness to refer the patient for investigation. The greater incidence of lung cancer amongst the CXRs performed in Leeds is possibly due to an incidence of lung cancer higher that the national average of within the region.^15^

Specific clinical indications for CXR and whether the examination was undertaken due to explicit concern regarding possible lung cancer were not evaluated. However, this reflects routine clinical practice and lung cancer is incidentally detected on CXRs with no suspicion of malignancy indicated by the referring clinician. In addition, the symptoms defined by NICE as indicating possible lung cancer are shared by a range of benign conditions, and there is no validated means to reliably determine the underlying concerns of the referring clinician in retrospect. It is likely that analysis restricted to referrals with an explicitly stated concern for cancer, and/or specific symptoms would lead to a higher cancer incidence but at the risk of missing a proportion of lung cancer cases, but this requires additional evaluation. Similarly, it would be desirable to have been able to have performed a more detailed evaluation the impact of smoking, however this smoking status information was only recorded in the broadly defined categories outlined.

The reference standard used is the diagnosis of lung cancer within 2 years. The standard assumes that the malignancy is present for the entire time-period and would present clinically within that time. The timeframe is based upon the estimated mean sojourn time for lung cancer which is the average time that malignancies are thought to be detectable before their clinical presentation. Modelling derived from CXR and CT screening studies of non-small cell lung cancer, the commonest sub-type of lung cancer, consistently place the minimal estimate of the sojourn time at over 2-yrs and the standard has previously been used in published studies of diagnostic accuracy.^14^^,^^16–19^

## Conclusions

The study indicates that the national incidence of lung cancer amongst GP CXR referrals within the United Kingdom is 1.4 % although the rate may increase in regions with a high incidence of lung cancer. A simple stratification of risk can be achieved using age and smoking status, with some adjustment for local incidence. The information may be used to provide a rationale for when to conduct a further evaluation for lung cancer with CT without initial CXR or despite negative initial investigations. Centres should consider including questions regarding smoking status within the CXR requesting process to both determine their local incidence of lung cancer amongst CXR referrals and as a method of triage of those at high risk.

## Supplementary Material

tqae142_Supplementary_Data
