# Supplementary material for: The incidence of lung cancer amongst primary care chest radiograph referrals—an evaluation of national and local datasets within the United Kingdom
Source: Br J Radiol. 2024 Aug 20;97(1163):1769–74. doi: 10.1093/bjr/tqae142 (PMC11491611; doi:10.1093/bjr/tqae142)
Supplement: tqae142_Supplementary_Data [file tqae142_supplementary_data.docx]

| CPRD per CXR | All | | | | current-smoker | | | | ex-smoker | | | | never-smoker | | | |
| --- | --- | --- | --- | --- | --- | --- | --- | --- | --- | --- | --- | --- | --- | --- | --- | --- |
| Age | Number | Cancers | 2-yr incidence | 95% CI | Number | Cancers | 2-yr incidence | 95% CI | Number | Cancers | 2-yr incidence | 95% CI | Number | Cancers | 2-yr incidence | 95% CI |
| 40-44 | 11,190 | 8 | 0.1% | 0.0-0.1 | 3,518 | 4 | 0.1% | 0.0-0.3 | 3,141 | 2 | 0.1% | 0.0-0.2 | 3,945 | 1 | 0.0% | 0.0-0.1 |
| 45-49 | 15,909 | 27 | 0.2% | 0.1-0.3 | 5,086 | 15 | 0.3% | 0.2-0.5 | 4,753 | 7 | 0.1% | 0.1-0.3 | 5,435 | 5 | 0.1% | 0.0-0.2 |
| 50-54 | 21,467 | 99 | 0.5% | 0.4-0.6 | 7,049 | 55 | 0.8% | 0.6-1.0 | 6,574 | 40 | 0.6% | 0.4-0.8 | 7,094 | 0 | 0.0% | 0.0-0.1 |
| 55-59 | 25,052 | 188 | 0.8% | 0.7-0.9 | 7,669 | 112 | 1.5% | 1.2-1.8 | 8,565 | 62 | 0.7% | 0.6-0.9 | 8,015 | 13 | 0.2% | 0.1-0.3 |
| 60-64 | 26,614 | 287 | 1.1% | 1.0-1.2 | 7,427 | 164 | 2.2% | 1.9-2.6 | 10,441 | 96 | 0.9% | 0.8-1.1 | 7,971 | 21 | 0.3% | 0.2-0.4 |
| 65-69 | 29,296 | 451 | 1.5% | 1.4-1.7 | 6,888 | 227 | 3.3% | 2.9-3.7 | 13,718 | 201 | 1.5% | 1.3-1.7 | 7,857 | 16 | 0.2% | 0.1-0.3 |
| 70-74 | 31,942 | 695 | 2.2% | 2.0-2.3 | 6,157 | 282 | 4.6% | 4.1-5.1 | 16,971 | 362 | 2.1% | 1.9-2.4 | 8,016 | 29 | 0.4% | 0.2-0.5 |
| 75-79 | 26,262 | 608 | 2.3% | 2.1-2.5 | 4,136 | 217 | 5.2% | 4.6-5.8 | 14,743 | 360 | 2.4% | 2.2-2.7 | 6,721 | 27 | 0.4% | 0.3-0.6 |
| 80- | 41,123 | 842 | 2.0% | 1.9-2.2 | 4,121 | 197 | 4.8% | 4.2-5.5 | 22,554 | 567 | 2.5% | 2.3-2.7 | 12,668 | 67 | 0.5% | 0.4-0.7 |
| Total | 228,855 | 3,205 | 1.4% | 1.4-1.5 | 52,051 | 1,273 | 2.4% | 2.3-2.6 | 101,460 | 1,697 | 1.7% | 1.6-1.8 | 67,722 | 179 | 0.3% | 0.2-0.3 |
| LTHT per CXR | All | | | | current-smoker | | | | ex-smoker | | | | never-smoker | | | |
| 40-44 | 4267 | 2 | 0.0% | 0.0-0.2 | 1180 | 0 | 0.0% | 0.0-0.3 | 928 | 1 | 0.1% | 0.0-0.6 | 1984 | 1 | 0.1% | 0.0-0.3 |
| 45-49 | 5554 | 10 | 0.2% | 0.0-0.3 | 1643 | 6 | 0.4% | 0.1-0.8 | 1343 | 3 | 0.2% | 0.1-0.7 | 2321 | 1 | 0.0% | 0.0-0.2 |
| 50-54 | 6828 | 38 | 0.6% | 0.4-0.8 | 1952 | 21 | 1.1% | 0.7-1.7 | 1629 | 12 | 0.7% | 0.4-1.3 | 2717 | 3 | 0.1% | 0.0-0.3 |
| 55-59 | 7343 | 65 | 0.9% | 0.7-1.1 | 1871 | 38 | 2.0% | 1.4-2.8 | 2023 | 16 | 0.8% | 0.5-1.3 | 2875 | 8 | 0.3% | 0.1-0.6 |
| 60-64 | 7485 | 106 | 1.4% | 1.2-1.7 | 1536 | 54 | 3.5% | 2.7-4.6 | 2467 | 33 | 1.3% | 0.9-1.9 | 2823 | 7 | 0.2% | 0.1-0.5 |
| 65-69 | 8438 | 161 | 1.9% | 1.6-2.2 | 1430 | 68 | 4.8% | 3.7-6.0 | 3295 | 72 | 2.2% | 1.7-2.7 | 2987 | 7 | 0.2% | 0.1-0.5 |
| 70-74 | 7822 | 181 | 2.3% | 2.0-2.7 | 1083 | 57 | 5.3% | 4.0-6.8 | 3479 | 92 | 2.6% | 2.1-3.2 | 2638 | 15 | 0.6% | 0.3-0.9 |
| 75-79 | 6189 | 186 | 3.0% | 2.6-3.5 | 637 | 56 | 8.8% | 6.7-11.3 | 2832 | 104 | 3.7% | 3.0-4.4 | 2290 | 11 | 0.5% | 0.2-0.9 |
| 80- | 8513 | 219 | 2.6% | 2.3-2.9 | 466 | 40 | 8.6% | 6.2-11.5 | 3808 | 123 | 3.2% | 2.7-3.8 | 3774 | 46 | 1.2% | 0.9-1.6 |
| Total | 62439 | 968 | 1.6% | 1.5-1.7 | 11798 | 340 | 2.9% | 2.6-3.2 | 21804 | 456 | 2.1% | 1.9-2.3 | 24409 | 99 | 0.4% | 0.3-0.5 |

Appendix 1. Table of the incidence of lung cancer within 2-yrs per CXR for CPRD and LTHT by smoking status. Events with unknown smoking status are not listed.

| CPRD per person | All | | | | current-smoker | | | | | | ex-smoker | | | | | | never-smoker | | | | | |
| --- | --- | --- | --- | --- | --- | --- | --- | --- | --- | --- | --- | --- | --- | --- | --- | --- | --- | --- | --- | --- | --- | --- |
| Age | Number | Cancers | 2-yr incidence | 95% CI | Number | Cancers | | 2-yr incidence | 95% CI | Number | | Cancers | | 2-yr incidence | 95% CI | Number | | Cancers | | 2-yr incidence | 95% CI |  |
| 40-44 | 9552 | 7 | 0.1% | 0.0-0.1 | 2,987 | 3 | | 0.1% | 0.0-0.3 | 2,688 | | 2 | | 0.1% | 0.0-0.3 | 3,357 | | 1 | | 0.0% | 0.0-0.2 |  |
| 45-49 | 13479 | 22 | 0.2% | 0.1-0.3 | 4,215 | 14 | | 0.3% | 0.2-0.6 | 4,008 | | 5 | | 0.1% | 0.0-0.3 | 4,692 | | 3 | | 0.1% | 0.0-0.2 |  |
| 50-54 | 17851 | 75 | 0.4% | 0.3-0.5 | 5,717 | 43 | | 0.8% | 0.5-1.0 | 5,446 | | 30 | | 0.6% | 0.4-0.8 | 6,023 | | 0 | | 0.0% | 0.0-0.1 |  |
| 55-59 | 20437 | 128 | 0.6% | 0.5-0.7 | 6,070 | 82 | | 1.4% | 1.1-1.7 | 7,002 | | 38 | | 0.5% | 0.4-0.7 | 6,673 | | 7 | | 0.1% | 0.0-0.2 |  |
| 60-64 | 21335 | 218 | 1.0% | 0.9-1.2 | 5,800 | 128 | | 2.2% | 1.8-2.6 | 8,298 | | 69 | | 0.8% | 0.7-1.1 | 6,571 | | 15 | | 0.2% | 0.1-0.4 |  |
| 65-69 | 22918 | 345 | 1.5% | 1.4-1.7 | 5,218 | 175 | | 3.4% | 2.9-3.9 | 10,699 | | 153 | | 1.4% | 1.2-1.7 | 6,302 | | 12 | | 0.2% | 0.1-0.3 |  |
| 70-74 | 24456 | 512 | 2.1% | 1.9-2.3 | 4,566 | 210 | | 4.6% | 4.0-5.3 | 12,850 | | 262 | | 2.0% | 1.8-2.3 | 6,371 | | 20 | | 0.3% | 0.2-0.5 |  |
| 75-79 | 19720 | 439 | 2.2% | 2.0-2.4 | 2,975 | 158 | | 5.3% | 4.5-6.2 | 11,012 | | 257 | | 2.3% | 2.1-2.6 | 5,191 | | 21 | | 0.4% | 0.3-0.6 |  |
| 80- | 31036 | 591 | 1.9% | 1.8-2.1 | 3,050 | 147 | | 4.8% | 4.1-5.6 | 16,769 | | 387 | | 2.3% | 2.1-2.6 | 9,769 | | 49 | | 0.5% | 0.4-0.7 |  |
| Total | 180784 | 2337 | 1.3% | 1.2-1.4 | 40,598 | 960 | | 2.4% | 2.2-2.5 | 78,772 | | 1,203 | | 1.5% | 1.4-1.6 | 54,949 | | 128 | | 0.2% | 0.2-0.3 |  |
|  |  |  |  |  |  |  | |  |  |  | |  | |  |  |  | |  | |  |  |  |
| LTHT per person | All | | | | current-smoker | | | | | | ex-smoker | | | | | | never-smoker | | | | | |
| 40-44 | 3838 | 0 | 0.0% | 0.0-0.1 | 1060 | | 0 | 0.0% | 0.0-0.3 | 821 | | | 1 | 0.1% | 0.0-0.7 | 1797 | | | 1 | 0.1% | 0.0-0.3 |  |
| 45-49 | 4820 | 9 | 0.2% | 0.1-0.4 | 1395 | | 6 | 0.4% | 0.2-0.9 | 1151 | | | 2 | 0.2% | 0.0-0.6 | 2054 | | | 1 | 0.0% | 0.0-0.3 |  |
| 50-54 | 5902 | 33 | 0.6% | 0.4-0.8 | 1626 | | 17 | 1.0% | 0.6-1.7 | 1414 | | | 11 | 0.8% | 0.4-1.4 | 2384 | | | 3 | 0.1% | 0.0-0.3 |  |
| 55-59 | 6151 | 54 | 0.9% | 0.7-1.1 | 1498 | | 33 | 2.2% | 1.5-3.1 | 1691 | | | 15 | 0.9% | 0.5-1.5 | 2450 | | | 5 | 0.2% | 0.1-0.5 |  |
| 60-64 | 6106 | 88 | 1.4% | 1.2-1.8 | 1233 | | 46 | 3.7% | 2.7-5.0 | 1969 | | | 26 | 1.3% | 0.9-1.9 | 2336 | | | 6 | 0.3% | 0.1-0.6 |  |
| 65-69 | 6710 | 123 | 1.8% | 1.5-2.2 | 1124 | | 47 | 4.2% | 3.1-5.5 | 2570 | | | 59 | 2.3% | 1.8-3.0 | 2408 | | | 5 | 0.2% | 0.1-0.5 |  |
| 70-74 | 6059 | 148 | 2.4% | 2.1-2.9 | 818 | | 47 | 5.7% | 4.3-7.6 | 2615 | | | 73 | 2.8% | 2.2-3.5 | 2099 | | | 12 | 0.6% | 0.3-1.0 |  |
| 75-79 | 4761 | 145 | 3.0% | 2.6-3.6 | 493 | | 43 | 8.7% | 6.4-11.6 | 2135 | | | 79 | 3.7% | 2.9-5.0 | 1779 | | | 9 | 0.5% | 0.2-1.0 |  |
| 80+ | 6602 | 166 | 2.5% | 2.2-2.9 | 358 | | 30 | 8.4% | 5.7-11.8 | 2879 | | | 88 | 3.1% | 2.5-3.8 | 2975 | | | 38 | 1.3% | 0.9-1.8 |  |
| Total | 50949 | 768 | 1.5% | 1.4-1.6 | 9605 | | 269 | 2.8% | 2.5-3.2 | 17245 | | | 354 | 2.1% | 1.9-2.3 | 20282 | | | 80 | 0.4% | 0.3-0.5 |  |

Appendix 2. Incidence of lung cancer within 2-yrs per individual by age and smoking status from CPRD and LTHT-RIS data. Events with unknown smoking status are not listed.

|  | **Men** | | | **Women** | | |
| --- | --- | --- | --- | --- | --- | --- |
| **Age** | **current smokers** | **ex-smokers** | **never smokers** | **current smokers** | **ex-smokers** | **never smokers** |
| 40-44 | 0.17% | 0.15% | 0.06% | 0.06% | 0.00% | 0.00% |
| 45-49 | 0.30% | 0.23% | 0.22% | 0.29% | 0.08% | 0.00% |
| 50-54 | 0.90% | 0.69% | 0.00% | 0.67% | 0.54% | 0.00% |
| 55-59 | 1.62% | 0.41% | 0.11% | 1.33% | 1.02% | 0.20% |
| 60-64 | 2.05% | 1.01% | 0.06% | 2.35% | 0.82% | 0.42% |
| 65-69 | 3.24% | 1.19% | 0.09% | 3.35% | 1.77% | 0.28% |
| 70-74 | 4.83% | 2.01% | 0.39% | 4.35% | 2.28% | 0.35% |
| 75-79 | 5.57% | 2.55% | 0.59% | 4.94% | 2.31% | 0.31% |
| 80- | 4.43% | 2.80% | 0.69% | 5.08% | 2.17% | 0.46% |
| Total | 2.50% | 1.75% | 0.25% | 2.40% | 1.59% | 0.27% |

Appendix 3. Incidence of lung cancer within 2-years per CXR for men and women (CPRD). Full gender analysis of per person incidence and results from the LTHT-RIS evaluation are not shown.
